# Silicon Dioxide Nanoparticles Enhance Endotoxin-Induced Lung Injury in Mice

**DOI:** 10.3390/molecules23092247

**Published:** 2018-09-03

**Authors:** Je-Won Ko, Hae-Jun Lee, Na-Rae Shin, Yun-Soo Seo, Sung-Ho Kim, In-Sik Shin, Joong-Sun Kim

**Affiliations:** 1 College of Veterinary Medicine (BK21 Plus Project Team), Chonnam National University, 77 Yongbong-ro, Buk-gu, Gwangju 61186, Korea; rheoda@gmail.com (J.-W.K.); tlsskfo870220@gmail.com (N.-R.S.); shokim@chonnam.ac.kr (S.-H.K.); 2 Division of Radiation Biomedical Research, Korea Institute of Radiological & Medical Sciences, Seoul 01812, Korea; hjlee@kirams.re.kr; 3 Herbal Medicinal Research Center, Korea Institute of Oriental Medicine, 1672 Yuseong-daero, Yuseong-gu, Daejeon 34054, Korea; sys0109@kiom.re.kr

**Keywords:** silicon dioxide nanoparticles, respiratory tract, inflammation, mitogen-activated protein kinase

## Abstract

Silicon dioxide nanoparticles (SiONPs), which are metal oxide nanoparticles, have been used in a wide variety of applications. In this study, acute pulmonary responses were examined after the intranasal instillation of SiONPs in mice primed with or without lipopolysaccharide (LPS, intranasal, 5 µg/mouse). The exposure to SiONPs increased the inflammatory cell counts and proinflammatory cytokines in the bronchoalveolar lavage fluid. SiONPs induced airway inflammation with increases in the phosphorylation of mitogen-activated protein kinases (MAPKs). The ratios of the inflammatory responses induced by the SiONPs were increased in the acute pulmonary disease model primed by LPS. Taken together, SiONPs exhibited toxicity to the respiratory system, which was associated with MAPK phosphorylation. In addition, the exposure to SiONPs exacerbated any existing inflammatory pulmonary diseases. These data showed the additive, as well as synergistic, interaction effects of SiONPs and LPS. We conclude that the exposure to SiONPs causes potential toxicity in humans, especially those with respiratory diseases.

## 1. Introduction

Nanotechnology is widely used in various industries, such as electronic, biosensor, drug delivery and DNA vaccine adjuvant industries [1]. In particular, silicon dioxide nanoparticles (SiONPs) are used in a wide variety of applications [2,3]. However, the potential toxicity issues arising from the use of these powerful nanoparticles are often ignored [4]. Fortunately, toxicity studies have been carried out recently as the use of SiONPs has increased. Previous studies have demonstrated that SiONPs can cause different toxic effects, including lipid peroxidation, oxidative stress, oxidative DNA damage, disruption of the cell membrane, apoptosis and reduced proliferation [5,6]. In particular, according to Hassankhani et al. [7], the target organs of SiONPs are the lung, liver, testis and kidney. Besides, considering that the main route of exposure to SiONPs is the respiratory tract, a pulmonary toxicity test must be thoroughly performed [8].

Asian dust, which originates from the lands of China and Mongolia during the springtime, is a yellow sand dust that is an important threat to human health in East Asian countries, such as Korea, Japan and China [9]. These particles can easily reach the respiratory tract and more importantly, they can aggravate symptoms in patients that already have respiratory diseases [10]. According to Schwartz [11], the epidemiologic data demonstrated that there was a deterioration in the symptoms of patients with preexisting inflammatory lung diseases. Asian dust includes many pollutant materials, especially particulate matter less than 10 μm in diameter, which can reach small airway bronchioles [9]. The most abundant component in the Asian sand dust is SiONPs (52.3%) [12]. The exposure to Asian dust is difficult to avoid for people with respiratory diseases because they inevitably encounter it in their daily lives. For this reason, there is the need to carry out tests for the exacerbated toxicity of SiONP exposure in combination with existing inflammatory lung diseases as well as a toxicity evaluation of a single exposure to SiONPs.

In the present study, the acute toxicity of SiONPs was examined in the lung after intranasal instillation. Furthermore, the pulmonary response of lungs primed with lipopolysaccharide (LPS) to SiONPs was also determined. In particular, the effects of intranasal exposure to SiONPs on inflammatory signaling pathways and transcription factor activation in lungs were determined both in the absence and presence of LPS.

## 2. Results

### 2.1. Physicochemical Characterization of SiONPs

**Figure 1:**
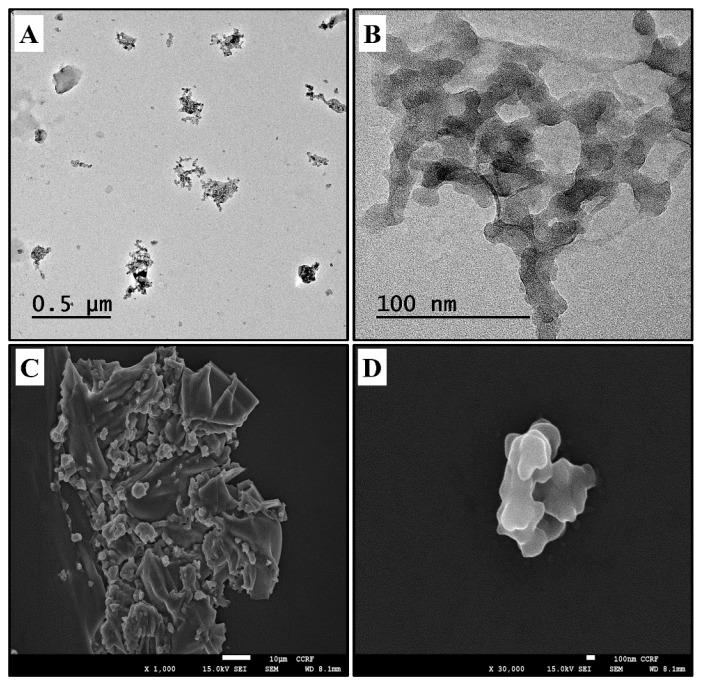
Morphology of SiONPs. (**A**,**B**) TEM images of SiONPs. (**C**,**D**) SEM images of SiONPs.

The transmission electron microscope (TEM) and scanning electron microscope (SEM) morphologies of the SiONPs are shown in Figure 1, which indicated the aggregation of the particles. The analysis of the vehicle-based solution of the SiONPs by TEM indicated a size distribution of 50 nm, with a mean and SD of 46.67 ± 6.85%.

### 2.2. Effects of SiONPs on Inflammatory Cell Counts in the bronchoalveolar lavage fluid (BALF) from Vehicle- and LPS-Treated Mice

**Figure 2:**
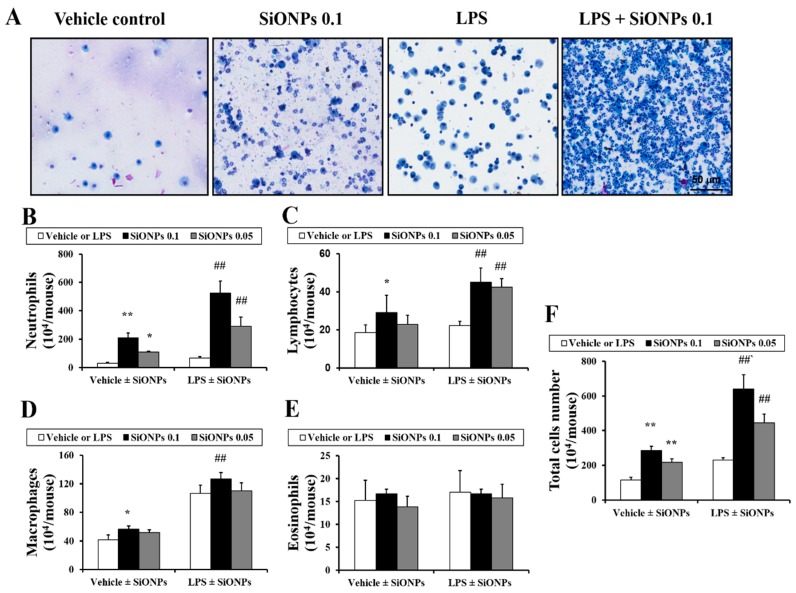
Effects of SiONPs exposure on inflammatory cell counts in BALF. (**A**) Representative images of inflammatory cells of each group. (**B**) Neutrophils in BALF of each group. (**C**) Lymphocytes in BALF of each group. (**D**) Macrophages in BALF of each group. (**E**) Eosinophils in BALF of each group. (**F**) Total cells in BALF of each group. Data represent the means ± SD (*n* = 5 per group). * *p* < 0.05 and ** *p* < 0.01 vs. vehicle control. ## *p* < 0.01 vs. LPS.

In the vehicle ± SiONPs group, the exposure to the SiONPs caused a mild increase in the number of lymphocytes, neutrophils and macrophages in the BALF in a dose-dependent manner compared to those of the vehicle only-treated mice (Figure 2). In the LPS ± SiONPs group, the exposure to the SiONPs significantly increased the number of lymphocytes, neutrophils and macrophages in the BALF compared to those of the LPS only-treated mice. However, the number of eosinophils in the BALF did not show a significant change in both groups. The LPS only-treated mice exhibited significant increases in the numbers of neutrophils, macrophages and total cells compared to those of the vehicle only-treated mice. Comparing the results between the vehicle or LPS ± SiONPs groups, the LPS ± SiONPs-treated mice had a statistically significant increase in the number of neutrophils, lymphocytes, macrophages and total cells compared to the vehicle ± SiONPs-treated mice.

### 2.3. Effects of SiONPs on Inflammatory Cytokines in the BALF from the Vehicle- and LPS Treated-Mice

**Figure 3:**
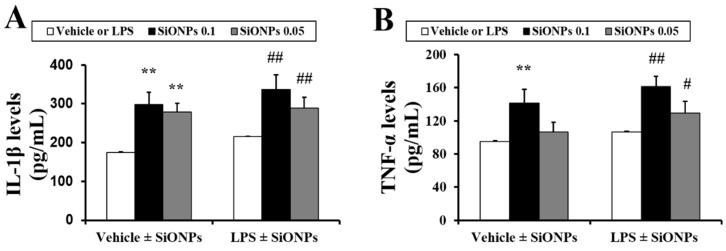
Effect of SiONPs exposure on proinflammatory cytokines in BALF. Levels of (**A**) IL-1β and (**B**) TNF-α. Data represent the means ± SD (*n* = 5 per group). ** *p* < 0.01 vs. vehicle control. # *p* < 0.01 and ## *p* < 0.05 vs. LPS.

In the vehicle ± SiONPs group, the exposure to the SiONPs caused a significant increase in the production of interleukin (IL)-1β and tumor necrosis factor (TNF)-α in the BALF in a dose-dependent manner compared to those of the vehicle only-treated mice (Figure 3A,B, respectively). In the LPS ± SiONPs group, the exposure to the SiONPs increased the IL-1β and TNF-α production in the BALF compared to the LPS only-treated mice. Comparing the results between the vehicle and LPS ± SiONPs groups, the LPS ± SiONPs groups had an elevated production of cytokines in comparison to the vehicle ± SiONPs-treated group although this difference was not significant.

### 2.4. Effects of SiONPs on Histological Alteration in the Vehicle- and LPS-Treated Mice

**Figure 4:**
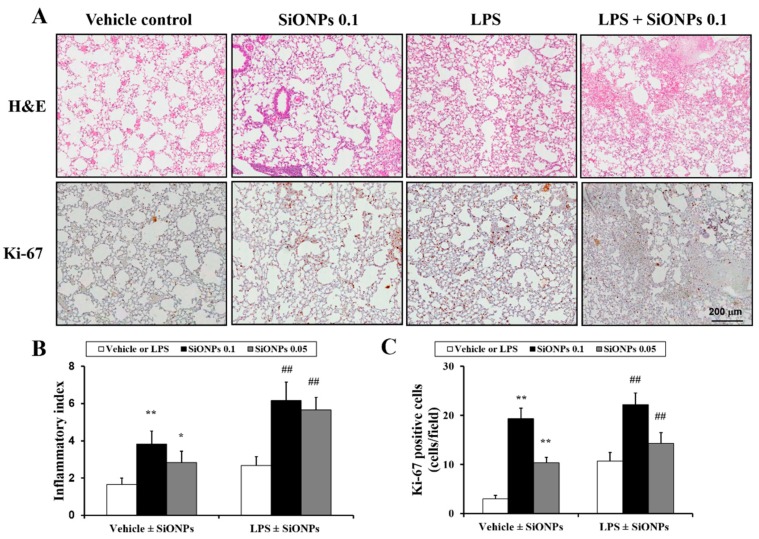
Effect of SiONPs exposure on inflammatory responses in lung tissues of each group. (**A**) Representative images of inflammatory responses in lung tissue of each group. (**B**) Quantitative analysis of inflammatory responses in lung tissue of each group. (**C**) Quantitative analysis of Ki-67 expression in lung tissue of each group. Data represent the means ± SD (*n* = 5 per group). * and *p* < 0.05 and ** *p* < 0.01 vs. vehicle control. ## *p* < 0.01 vs. LPS.

In the vehicle ± SiONPs group, the inflammation of the lung tissue increased dose-dependently in the mice exposed to SiONPs compared to the mice treated only with the vehicle. In particular, at a high dose of SiONPs, extensive inflammation was observed in the lung tissue (Figure 4A,B). In the LPS ± SiONPs group, the mice exposed to SiONPs showed a marked increase in airway inflammation compared to the mice treated only with LPS. LPS only-treated mice demonstrated an increase in the inflammatory index compared to the vehicle only-treated mice. Comparing the results between the vehicle and LPS ± SiONPs groups, the LPS ± SiONPs groups had a statistically significant increase in the inflammatory index compared to that of the vehicle ± SiONPs-treated mice. In addition, Ki-67 expression was increased in LPS ± SiONPs groups compared with the LPS only-treated group.

### 2.5. Effects of SiO2 on MAPK Expression in LPS-Treated Mice

**Figure 5:**
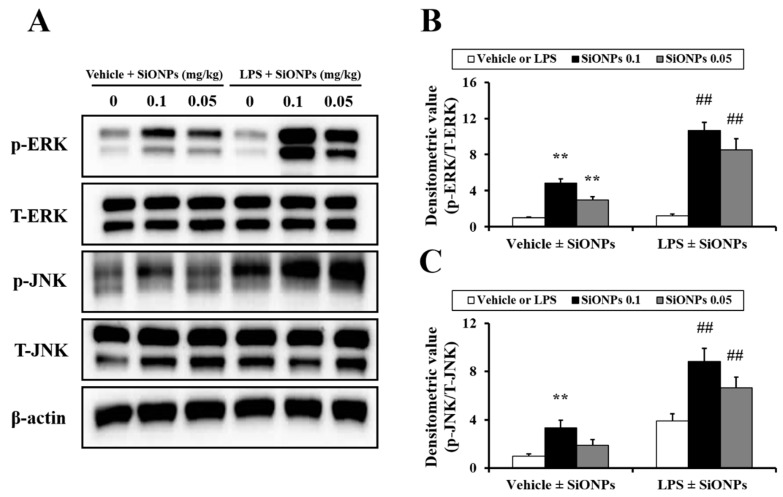
Effect of SiONPs exposure on ERK and JNK phosphorylation in lung tissues of each group. (**A**) ERK and JNK expression on gels. (**B**) Densitometric value of ERK phosphorylation. (**C**) Densitometric value of JNK phosphorylation. Data represent the means ± SD (*n* = 5 per group). ** *p* < 0.01 vs. vehicle control. ## *p* < 0.01 vs. LPS.

In the vehicle ± SiONPs group, the exposure to SiONPs increased the phosphorylation of extracellular signal-regulated kinase (ERK) and c-Jun amino-terminal kinase (JNK) in the lung tissue compared to the vehicle only-treated mice, which occurred in a dose-dependent manner (Figure 5). In the LPS ± SiONPs group, the exposure to SiONPs further elevated the phosphorylation of ERK and JNK compared to that of the LPS only-treated mice. These ratios of elevation were larger that than those observed for the SiONPs only-treated mice. Comparing the results between the two groups, the ratio of the increase in the phosphorylation of ERK and JNK due to the treatment with SiONPs was found to be greater in the LPS ± SiONPs group compared to the vehicle ± SiONPs group. In particular, the phosphorylation of JNK in the LPS ± SiONPs group increased significantly compared to that in the vehicle ± SiONPs-treated group.

## 3. Discussion

SiONPs have recently been used in many industrial fields [13]. They also comprise a large proportion of Asian sand dust, which may be a problem for patients with respiratory diseases [10,14]. Therefore, experiments must be performed to determine the effects of the respiratory toxicity and the exacerbation of respiratory pulmonary diseases due to SiONPs. In the present study, we evaluated the toxicological role of SiONPs, with a focus on pulmonary inflammation and the exacerbation of inflammation, in an acute pulmonary mouse model induced by LPS. Our results demonstrated that the exposure to SiONPs markedly elevated the inflammatory cell counts and inflammatory mediators, which was consistent with the histopathological evidence, including inflammatory cell accumulation. Furthermore, the exposure to SiONPs increased the phosphorylation of ERK and JNK. These increases were significant in the LPS-induced pneumonia model compared with the mice treated only with the SiONPs.

In this study, we injected LPS at a dose of 5 µg/mouse (250 µg/kg) to induce lung injury. In our previous study, we injected 10 µg/mouse (500 µg/kg), which is considered to be a reasonable dose, to induce acute lung injury. After this, we carried out an autopsy 24 h later when most cytokines are thought to be activated [1,15,16]. The reason for choosing the LPS concentration to 5 µg/mouse in this manuscript is due to the need to ensure that we used a sufficient dose to induce the acute lung injury and simultaneously consider the additional damage caused by SiONPs. The reason for the instillation time of SiONPs (24 h after LPS instillation) is that the LPS-induced acute lung injury state is most evident at this time point. Therefore, we thought that SiONPs should be instilled at this point to determine whether SiONPs would exacerbate existing lung diseases. In addition, the autopsy was performed 48 h after the instillation of SiONPs as this is considered to be the best time to judge toxicity by nanoparticles according to our previous study [17].

It has been well established that the lung is the main target organ of SiONPs. SiONPs at a size of 20 and 80 nm can accumulate in the lung when intravenously injected [18] and subsequently induce histopathological alterations, such as interstitial pneumonia, increased thickness of the alveolar wall and bronchopneumonia in the lungs [7]. In particular, the exposure to SiONPs induced airway inflammation, with increases in the number of inflammatory cells and in the production of proinflammatory cytokines [19]. Of the inflammatory cells, neutrophils have a crucial role in the development of inflammation [17]. Neutrophils contain a variety of stimulant mediators, which mediate cytokines and tissue lysis enzymes that cause problems associated with pulmonary function, including lung inflammation and deterioration [20]. Our findings are similar to the results obtained in previous studies. The exposure to SiONPs induced an increase in the number of inflammatory cells in the BALF, especially neutrophils, with an accompanying elevation in the proinflammatory cytokines, including IL-1β and TNF-α. Furthermore, SiONPs increased the infiltration of inflammatory cells in the lung tissues of mice. These alterations were prominently observed in the LPS-treated groups. LPS ± SiONPs-treated mice had significantly elevated inflammatory cell counts, cytokines and lung inflammation compared to the mice treated only with LPS, for which there were greater increases than in the vehicle ± SiONPs-treated mice. Based on these results, the exposure to SiONPs induced airway inflammation with increases in inflammatory mediators, which was further aggravated in the presence of a LPS-induced acute pulmonary injury. Therefore, these results indicate that pulmonary inflammation, which was caused by the exposure to SiONPs, worsens in the presence of pulmonary disease.

It is well known that MAPKs play important roles in the inflammatory response, including by being involved in the production of proinflammatory cytokines and chemokines through the regulation of the downstream signaling pathways [21,22]. Various stimuli, such as cytokines, growth factors and LPS, may trigger the phosphorylation of MAPKs, including ERK and JNK, to mediate proinflammatory gene transcription [23]. Of these stimuli, nanoparticles can trigger cell injury through reactive oxygen species-mediated MAPK signaling [24]. According to previous studies, a number of nanoparticles, including copper oxide nanoparticles, gold nanoparticles and silver nanoparticles, induces the phosphorylation of MAPKs [1,25,26]. In a pulmonary toxicity study, Park et al. [17] reported that copper oxide nanoparticles aggravate airway inflammation in asthmatic mice via MAPK signaling. In particular, Christen and Fent [27] reported that SiONPs-induced ER stress and oxidative stress results in the production of proinflammatory cytokines via the phosphorylation of MAPKs, including ERK and JNK. In this study, the exposure to SiONPs increased the phosphorylation of ERK and JNK. Similar to the results for the production of the inflammatory cytokines, the increase in the phosphorylation of ERK and JNK, which was caused by the exposure to SiONPs, was found to be greater in the acute pulmonary disease models that were induced by LPS compared to the vehicle ± SiONPs-treated mice. Considering previous reports and our results, the elevation in the phosphorylation of JNK and ERK induced by the exposure to SiONPs was closely associated with marked increases in the pulmonary inflammation status in the presence of a LPS-induced acute pulmonary injury.

In conclusion, the respiratory tract exposure to SiONPs leads to inflammatory responses, including an increase in the number of inflammatory cells and production of proinflammatory cytokines. These alterations are more aggravated in the presence of a LPS-induced acute pulmonary injury than under normal conditions, which is related to the elevation of MAPK phosphorylation. Therefore, our results indicate that the exposure to SiONPs may be a greater risk factor for patients with pulmonary disease.

## 4. Materials and Methods

### 4.1. Nanoparticles

The tested articles examined in this study were commercially available SiONPs, which were purchased form Sigma Aldrich (St. Louis, MO, USA). Information provided by the manufacturer stated that the primary particle size, which was measured by transmission electron microscopy (TEM), was 50 nm. To quantify the size and morphology of the SiONPs, TEM characterization was performed using a JEM-1210 (JEOL, Tokyo, Japan), at an accelerating voltage of 200 kV. The samples were deposited on carbon-coated nickel grids and were air-dried overnight before TEM analysis. The samples for scanning electron microscopy (SEM) analysis were dispersed onto double-sided adhesive carbon tape on an aluminum SEM stub, before being dusted to release the loose particles.

### 4.2. Experimental Procedure for the Animal Experiments

Six-week-old female C57BL/6 mice (Central Lab. Animal Inc., Seoul, Korea) were used after one week of quarantine and acclimatization. The animals were maintained at 23 ± 2 °C in a room with a relative humidity of 50% ± 5%, artificial lighting from 08:00–20:00 and 13–18 air changes per hour. The mice were given a standard laboratory diet and water ad libitum. All experimental procedures were carried out in accordance with the NIH Guidelines for the Care and Use of Laboratory Animals and were conducted following a protocol approved by the Institutional Animal Care and Use Committee of Chonnam National University (CNU IACUC-YB-2016-18). The animals were cared for in accordance with the dictates of the National Animal Welfare Law of Korea.

The mice were divided into two groups, with each group divided into three subgroups as follows. The first was the vehicle ± SiONPs group, which received vehicle control (PBS instillation on days 1 to 4), 0.1 of SiONPs and 0.05 of SiONPs (PBS instillation on day 1; SiONPs instillation at doses of 0.1 mg/kg and 0.05 mg/kg on days 2–4, respectively). The second was the LPS ± SiONPs group, which received LPS (LPS instillation at a dose of 5 μg/mouse on day 1; PBS instillation on days 2–4), LPS + SiONPs 0.1 and LPS + SiONPs 0.05 (LPS instillation at a dose of 5 μg/mouse on day 1; SiONPs instillation at doses of 0.1 mg/kg and 0.05 mg/kg on days 2–4, respectively). All instillations were performed under slight anesthesia using an intraperitoneal injection of Zoletil 50^®^ (10 mg/kg; Virbac Laboratories, Carros, France). SiONPs were prepared in PBS and sonicated in an ultrasonicator (VCX-130, Spmcs and Materials, Newton, CT, USA) for 3 min (130 W, 20 kHZ, pulse 59/1) before intranasal instillation. The experiment schedule and treatment doses were determined by our previous studies [17,28].

The mice were sacrificed 48 h after the final instillation via an intraperitoneal injection of Zoletil 50**^®^** (Virbac Laboratories, Carros, France), and a tracheostomy was performed. To obtain the bronchoalveolar lavage fluid (BALF), ice-cold PBS (0.7 mL) was infused into the lungs twice and withdrawn each time using a tracheal cannula (a total volume of 1.4 mL). A 100-μL aliquot was used for the total cell count. After the exclusion of dead cells through trypan blue staining, the total inflammatory cell numbers were determined by counting the cells in at least five squares of a hemocytometer. To count differential inflammatory cells in the BALF, 100 μL of BALF was centrifuged (200 g, 4 °C, 10 min) onto slides using a cytospin (Hanil Science Industrial, Seoul, Korea). The slides were dried, fixed and stained with Diff-Quik**^®^** reagent (IMEB Inc., San Marcos, CA, USA) according to the manufacturer’s instructions. The remaining BALF was centrifuged (200 g, 4 °C, 10 min), and its supernatant was stored at −80 °C for the cytokine analysis.

### 4.3. Measurement of Proinflammatory Cytokines in the BALF

The levels of interleukin (IL)-1β and tumor necrosis factor-α (TNF-α) in the BALF were measured using commercial enzyme-linked immunosorbent assay (ELISA) kits (BD Biosciences, San Jose, CA, USA) according to the manufacturer’s protocol.

### 4.4. Histology

After the BALF samples were collected, the lung tissue was fixed using 10% (*v*/*v*) neutral buffered formalin. The tissues were paraffin-embedded, sectioned to have a section of 4 µm and stained using a hematoxylin and eosin (H&E) solution (Sigma-Aldrich, St. Louis, MO, USA) to estimate inflammation index. The quantitative analysis of the airway inflammation was conducted using image analysis software (IMT i-Solution software ver. 21.1, Vancouver, BC, Canada).

### 4.5. Measurement of Mitogen-Activated Protein Kinase (MAPK) Protein Expression in Lung Tissue

The lungs from each mouse were quickly and individually immersed in buffer H (50 mM glycerophosphate, 1.5 mM EGTA, 0.1 mM Na_3_VO_4_, 1 mM dithiothreitol, 10 g/mL aprotinin, 2 g/mL pepstatin, 10 g/mL leupeptin and 1 mM phenyl-methanesulfonylfluoride; pH 7.4), and sonicated for 10 s. The sample buffer of sodium dodecyl sulfate (SDS) was added to each homogenized sample, before the samples were heated at 100 °C for 10 min. After this, the samples were separated using 10% SDS–polyacrylamide gel electrophoresis (SDS–PAGE). The resolved proteins were transferred to a nitrocellulose membrane, which was blocked with 5% skim milk in PBS, containing 0.1% Tween-20 (PBST; pH 7.4), for 30 min at room temperature. The membrane was then incubated with the following primary antibodies in PBST overnight at 4 °C: extracellular signal-regulated kinases (ERK; 1:1000 dilution; Cell Signaling, Danvers, MA, USA), phospho-ERK (p-ERK; 1:1000 dilution; Cell Signaling, Shanghai, China); c-Jun N-terminal kinases (JNK; 1:1000 dilution; Santa Cruz, Dallas, TX, USA); p-JNK (1:1000 dilution; Santa Cruz, Dallas, TX, USA); and β-actin (1:1000; Cell Signaling, Shanghai, China). After extensive washing with PBST, the membrane was incubated with horseradish peroxidase-conjugated anti-rabbit or anti-mouse antibodies (1:10,000; Thermo Fisher Scientific, Rockford, IL, USA). Following this, the signals were visualized using a chemiluminescence kit (Thermo Fisher Scientific, Rockford, IL, USA). The membranes were stripped and re-probed with an anti-β-actin antibody (1:1000; Sigma–Aldrich, St. Louis, MO, USA) for normalization. Several exposure times were used to obtain the signals in the linear range. The bands were quantified using Scion Image Beta 4.0.2 for Windows XP software (Scion, Frederick, ME, USA).

### 4.6. Statistical Analysis

The data are reported as the mean ± standard deviation (SD). The data were analyzed using one-way analysis of variance, followed by the Student–Newman–Keuls post hoc test for multiple comparisons using GraphPad InStat software (GraphPad InStat, Inc., San Diego, CA, USA). In all cases, a *p* value < 0.05 was considered to be significant.
